# Chikungunya Virus Transmission Potential by Local *Aedes* Mosquitoes in the Americas and Europe

**DOI:** 10.1371/journal.pntd.0003780

**Published:** 2015-05-20

**Authors:** Anubis Vega-Rúa, Ricardo Lourenço-de-Oliveira, Laurence Mousson, Marie Vazeille, Sappho Fuchs, André Yébakima, Joel Gustave, Romain Girod, Isabelle Dusfour, Isabelle Leparc-Goffart, Dana L. Vanlandingham, Yan-Jang S. Huang, L. Philip Lounibos, Souand Mohamed Ali, Antoine Nougairede, Xavier de Lamballerie, Anna-Bella Failloux

**Affiliations:** 1 Institut Pasteur, Department of Virology, Arboviruses and Insect Vectors, Paris, France; 2 Sorbonne Universités, Université Pierre et Marie Curie-Paris 6, Institut de Formation Doctorale (IFD), Paris, France; 3 Laboratório de Transmissores de Hematozoários, Instituto Oswaldo Cruz, Fiocruz, Rio de Janeiro, Brazil; 4 Centre de Démoustication/Conseil Général de La Martinique, Fort-de-France, Martinique, France; 5 Agence Régionale de Danté (ARS) Guadeloupe, Saint-Martin et Saint-Barthélemy, Pole de Santé Publique, Gourbeyre, Guadeloupe, France; 6 Institut Pasteur de la Guyane, Unité d’Entomologie médicale, Cayenne, French Guiana; 7 Centre National de Référence (CNR) des Arbovirus, Institut de Recherche Biomédicale des Armées Hôpital d’Instruction des Armées Laveran, Marseille, France; 8 Department of Diagnostic Medicine and Pathobiology, College of Veterinary Medicine, Kansas State University, Manhattan, Kansas, United States of America; 9 Biosecurity Research Institute, Kansas State University, Manhattan, Kansas, United States of America; 10 Florida Medical Entomology Laboratory, University of Florida, Vero Beach, Florida, United States of America; 11 Aix Marseille Université, IRD French Institute of Research for Development, EHESP French School of Public Health, EPV UMR_D 190 ‘Emergence des Pathologies Virales’, Marseille, France; 12 IHU Méditerranée Infection, APHM Public Hospitals of Marseille, Marseille, France; University of Texas Medical Branch, UNITED STATES

## Abstract

**Background:**

Chikungunya virus (CHIKV), mainly transmitted in urban areas by the mosquitoes *Aedes aegypti* and *Aedes albopictus*, constitutes a major public health problem. In late 2013, CHIKV emerged on Saint-Martin Island in the Caribbean and spread throughout the region reaching more than 40 countries. Thus far, *Ae*. *aegypti* mosquitoes have been implicated as the sole vector in the outbreaks, leading to the hypothesis that CHIKV spread could be limited only to regions where this mosquito species is dominant.

**Methodology/Principal Findings:**

We determined the ability of local populations of *Ae*. *aegypti* and *Ae*. *albopictus* from the Americas and Europe to transmit the CHIKV strain of the Asian genotype isolated from Saint-Martin Island (CHIKV_SM) during the recent epidemic, and an East-Central-South African (ECSA) genotype CHIKV strain isolated from La Réunion Island (CHIKV_LR) as a well-characterized control virus. We also evaluated the effect of temperature on transmission of CHIKV_SM by European *Ae*. *albopictus*. We found that (i) *Aedes aegypti* from Saint-Martin Island transmit CHIKV_SM and CHIKV_LR with similar efficiency, (ii) *Ae*. *aegypti* from the Americas display similar transmission efficiency for CHIKV_SM, (iii) American and European populations of the alternative vector species *Ae*. *albopictus* were as competent as *Ae*. *aegypti* populations with respect to transmission of CHIKV_SM and (iv) exposure of European *Ae*. *albopictus* to low temperatures (20°C) significantly reduced the transmission potential for CHIKV_SM.

**Conclusions/Significance:**

CHIKV strains belonging to the ECSA genotype could also have initiated local transmission in the new world. Additionally, the ongoing CHIKV outbreak in the Americas could potentially spread throughout *Ae*. *aegypti*- and *Ae*. *albopictus*-infested regions of the Americas with possible imported cases of CHIKV to *Ae*. *albopictus*-infested regions in Europe. Colder temperatures may decrease the local transmission of CHIKV_SM by European *Ae*. *albopictus*, potentially explaining the lack of autochthonous transmission of CHIKV_SM in Europe despite the hundreds of imported CHIKV cases returning from the Caribbean.

## Author Summary

More than one million chikungunya cases have been reported in the Americas since October 2013, when the Asian genotype of chikungunya virus (CHIKV) was imported by a traveller returning from Asia. CHIKV is mainly transmitted in urban areas by the domestic mosquitoes *Aedes aegypti* and *Aedes albopictus*. In this study, we evaluate the potential for the CHIKV circulating in the Caribbean to initiate outbreaks in *Aedes*-infested regions of continental America and Europe by assessing the ability of local mosquitoes to experimentally transmit the virus. Mosquitoes were exposed to a blood-meal containing the virus which must overcome several barriers to infect various tissues in the vector before being secreted in the mosquito saliva when biting a host. We found that *Ae*. *aegypti* and *Ae*. *albopictus* transmitted similarly the virus. When exposing *Ae*. *albopictus* from Europe at a temperature of 20°C after infection, we detect a significant drop of CHIKV transmission potential. Our results suggest that the CHIKV outbreak in the Americas could potentially spread throughout *Ae*. *aegypti-* and *Ae*. *albopictus*-infested regions of the Americas however with a limited risk of spillovers in *Ae*. *albopictus*-infested regions in Europe. These data will be useful for adapting vector control strategies and epidemiological surveillance.

## Introduction

Chikungunya virus (CHIKV) is a mosquito-borne alphavirus that causes an acute febrile illness characterized by severe arthralgia. International travel and the global expansion of the two main CHIKV urban mosquito vectors, *Aedes aegypti* and *Aedes albopictus*, have enhanced the ability of the virus to spread to new regions where environmental conditions are permissive for viral transmission. Phylogenetically, CHIKV strains are classified into three distinct genotypes: Asian, West African, and Central/East/South African (ECSA). Over the past decade, the ECSA genotype significantly expanded its geographical range resulting in epidemics throughout India, Africa, Asia, and temperate Europe [1]. The ECSA genotype has also been repeatedly imported into the Americas, but autochthonous transmission has not been detected despite the ability of local vector species to transmit CHIKV [2,3]. Between 1995 and 2009, 109 imported CHIKV cases were identified in the United States alone, and among those, 13 (12%) developed a viremia high enough to infect mosquitoes [4]. Following the La Réunion epidemic in 2004, nine imported CHIKV cases were reported during 2006 in the French overseas departments of America [5].

In late 2013, the first locally-acquired CHIKV infections in the Americas were reported from Saint-Martin Island in the Caribbean [6,7]. The virus successively spread to other *Ae*. *aegypti*-infested islands. At the time of writing, this epidemic has caused more than 1,000,000 suspected cases in 43 countries from the Americas [8], with the potential for further spread to the rest of the continent. Surprisingly, the CHIKV strain responsible for this epidemic belongs to the Asian genotype [6] and not to the ECSA genotype as might have been predicted, based upon the high numbers of imported cases reported in recent years [4],[5]. Until now, only *Ae*. *aegypti* mosquitoes have been implicated in CHIKV transmission in the Americas [9]. Previous studies have suggested that the Asian genotype of CHIKV is constrained in its ability to adapt to *Ae*. *albopictus via* negative epistatic interactions of a single residue (E1-98T) with the E1-A226V substitution [10], which could limit viral spread to regions where this mosquito species is dominant [9]. Nevertheless, the potential role of *Ae*. *albopictus* as a vector in the Americas for the currently circulating CHIKV strain must also be considered. Indeed, *Ae*. *albopictus* is present in at least 19 countries in the Americas [11], and have previously been shown to experimentally transmit Asian strains of CHIKV [3].

This recent CHIKV epidemic in the Caribbean was also a threat for Europe. In France, from May 2 through July 4, 2014, the number of laboratory confirmed imported cases of CHIKV was much higher (126 cases) than in previous years [12]. This increase could potentially enhance the risk of local transmission in *Ae*. *albopictus*–infested European regions. Since its first report in Albania in 1979, *Ae*. *albopictus* has progressively spread and today is found in 20 European countries, causing a major public health concern [13]. Previous autochthonous transmissions of CHIKV in Italy and in France highlight the potential to establish transmission cycles involving temperate *Ae*. *albopictus* populations [14,15]. Furthermore, several studies revealed that European *Ae*. *albopictus* transmit ECSA CHIKV strains efficiently, at both 28°C and at lower temperatures [16,17].

Without a vaccine or specific treatment available, the only strategy for control of CHIKV outbreaks remains the suppression of vector populations, the use of individual protections (e.g. repellents) and the reinforcement of epidemiological surveillance in areas at high epidemic risk. The determination of vector competence in mosquito populations, defined as the ability of the vector to ingest, disseminate and transmit a pathogen, is essential in evaluating the risk of CHIKV transmission and spread into new areas as well as to design appropriate control strategies. Examination of virus population diversity by deep sequencing revealed strong bottlenecks when CHIKV passes mosquito anatomical barriers (midgut and salivary glands) leading to select variants with high epidemic potential in mosquito saliva [18]. Vector competence can be highly variable in natural populations and is determined by genotype-genotype interactions, in which successful transmission depends on some specific combination of mosquito and viral genetic characteristics [19], under specific environmental conditions [20]. Ambient temperatures and even daily fluctuations of temperature play a key role in shaping mosquito vector competence for pathogens [21,22]. Moreover, it has been shown that the potential of CHIKV transmission by *Ae*. *albopictus* strongly depends on the three-way combination of mosquito population, virus strain and temperature or Genotype x Genotype x Environment (G x G x E) interactions [17]. Additionally, knowledge about factors shaping vector capacity corresponding to the ability of a mosquito to act as a vector in the field (e.g., mosquito densities, mosquito trophic preferences, mosquito survival rate…) will be informative for a more accurate appraisal of CHIKV transmission. This study aims to evaluate the potential for the Asian CHIKV strain currently circulating in the Caribbean to initiate outbreaks in other countries of the Americas and to examine the effect of temperature on viral transmission in temperate Europe.

## Methods

### Ethics statement

The Institut Pasteur animal facility has received accreditation from the French Ministry of Agriculture to perform experiments on live animals in compliance with the French and European regulations on care and protection of laboratory animals. This study was approved by the Institutional Animal Care and Use Committee (IACUC) at the Institut Pasteur. No specific permits were required for the described field studies in locations which are not protected in any way and did not involve endangered or protected species.

### Mosquitoes

Eleven mosquito populations from the Caribbean, continental America and metropolitan France were used: eight populations of *Ae*. *aegypti* and three of *Ae*. *albopictus* (Table 1). The mosquitoes were field-collected in 2013 using ovitraps (10–58 per collection site). The field-collected eggs were immersed in water for hatching; larvae were reared at densities of 100–150 individuals per pan and fed with yeast tablets. Emerged adults were identified according to morphological criteria and maintained in cages at 28°±1°C with a 16h:8h light:dark cycle, 80% relative humidity, and supplied with a 10% sucrose solution. The F1 generation of mosquitoes was used for all infection assays except for *Ae*. *aegypti* from Saint-Martin (F3 generation) and from United States (F10 generation).

**Table 1:** Mosquito populations used.

Mosquito population	Collection site	Region	Country	Coordinates	Mosquito species used	Climate
USA	Vero Beach, Florida	North America	United States	27°35’N 80°22’W	AL	Subtropical
USA	Key West, Florida	North America	United States	24°33'N 81°46'W	AE	Subtropical
RIO	Jurujuba, Rio de Janeiro	South America	Brazil	22°55’S 43°07’W	AE/AL	Tropical
MACA	Macapá, Amapá	South America	Brazil	0°02' N 51°04' W	AE	Tropical
SMAR	Saint-Martin	Caribbean	France	18°04' N 63°03' W	AE	Tropical
SAIN	Les Saintes, Guadeloupe	Caribbean	France	15°51'N 61°34' W	AE	Tropical
GUAD	Abymes, Guadeloupe	Caribbean	France	16°16'N 61°30' W	AE	Tropical
MARTI	Vauclin, Martinica	Caribbean	France	14°34' N 60°51' W	AE	Tropical
CAYE	Cayenne, French Guiana	South America	France	4°55' N 52°19' W	AE	Tropical
FRA	Bar sur Loup, Metropolitan France	Europe	France	43°42' N 6°59' E	AL	Temperate

AE: *Ae*. *aegypti;* AL: *Ae*. *albopictus*.

Populations are listed according to their collection locality.

### Viral strains

Two CHIKV isolates of different genotypes were used: one CHIKV isolate from Saint-Martin Island belonging to the Asian lineage and one from La Réunion belonging to the East-Central-South African lineage. The isolate from La Réunion was the strain CHIKV 06.21 (CHIKV_LR) isolated in 2005 [23], and provided by the French National Reference Center for Arboviruses at the Institut Pasteur in Paris. The CHIKV strain from Saint-Martin was the CHIKV 20235 [6], isolated in 2013 from the serum of the first confirmed CHIKV local case in the New World. This strain was kindly provided by the French National Reference Center for Arboviruses in Marseille. The CHIKV_20235 (CHIKV_SM) is phylogenetically related to strains recently identified in Asia, *i*.*e*. China (2012), and the Philippines (2013), most of them sharing a specific four amino-acid deletion in the nsp3 gene [6]. The consensus sequences of CHIKV_LR and CHIKV_SM diverge by ~7% at the amino-acid level including two interesting changes at E1-226 (valine for CHIKV_LR and alanine for CHIKV_SM) [24] and E1-98 (alanine for CHIKV_LR and threonine for CHIKV_SM) [10]. The residue E1-98T exerts a negative epistatic interaction which blocks the ability of Asian strains to adapt to *Ae*. *albopictus via* the E1-A226V substitution [10]. None of the CHIKV strains harbor the E2 *Ae*. *albopictus*-adaptive mutations (E2-K252Q, E2-L210Q, E2-K233Q) described or predicted for CHIKV ECSA strains [25]. Stocks of CHIKV_LR were produced following three passages on *Ae*. *albopictus* C6/36 cells and CHIKV_SM was obtained after two passages on Vero cells. Supernatants were harvested and stored at -80°C until used for mosquito experimental infection assays. Viral titers were determined by serial 10-fold dilutions on Vero cells.

### Mosquito oral infections

Five to seven day-old female vectors were fed on an infectious blood-meal containing 1.4 mL of washed rabbit erythrocytes and 700 μL of viral suspension supplemented with a phagostimulant (ATP) at a final concentration of 5 mM. Four to six boxes of 60 mosquitoes were tested for each population. All 11 mosquito populations were challenged with CHIKV_LR and CHIKV_SM, each viral strain was provided separately in the blood meal. The titer of infectious blood-meals was 10^6.5^ pfu/mL in agreement with viremia levels detected in patients [26]. After the infectious blood-meal, fully engorged females were transferred to cardboard containers and maintained with 10% sucrose at 28°±1°C, a 16h:8h light:dark cycle and 80% humidity.

### Incubation temperature regimes

After infection, *Ae*. *albopictus* from Bar-sur-Loup in southeastern France (FRA in Table 1) were maintained in climatic chambers (KB 53, Binder, Tuttlingen, Germany) with a 16h:8h light:dark cycle under three different temperature regimes: (i) a constant temperature of 28°C ± 0.1°C, (ii) a constant temperature of 20°C±0.1°C or (iii) at temperatures displaying daily fluctuations between 17°C±0.1°C and 23°C± 0.1°C (average: 20°C±0·1°C). The constant temperature of 28°C was chosen both because it serves as a typical mean temperature in tropical regions and because this temperature is commonly used in our vector competence assays [3,27]. The constant temperature of 20°C and the temperature regime with daily fluctuations around 20°C were chosen as representative of the low-temperature threshold recorded during the Italian epidemic of CHIKV between June and September 2007 [14,28] and in southeast France in September 2010 (http://www.meteociel.fr) [15].

### Transmission analysis

Batches of ~20 mosquitoes of each combination of mosquito population-virus strain (and temperature regime for *Ae*. *albopictus* from Bar-sur-Loup, FRA in Table 1) were analyzed at days 3, 5 and 7 post-infection (pi) for the two CHIKV strains tested. Time-points were chosen based on the kinetics of CHIKV transmission efficiency obtained with mosquitoes from Rio de Janeiro, Brazil [3]. Additionally, mosquitoes from Saint-Martin (*Ae*. *aegypti*, SMAR) and from Bar-sur-Loup (*Ae*. *albopictus*, FRA) were also analyzed at day 2 pi to estimate the extrinsic incubation period [27]. To estimate viral transmission, saliva was collected from individual mosquitoes as described by Dubrulle and colleagues [27]. Briefly, wings and legs were removed from each mosquito and the proboscis was inserted into a 20 μL tip containing 5 μL of Fetal Bovine Serum (FBS). After 30 min of salivation, FBS containing saliva was expelled into 45 μL of Leibovitz L15 medium for further titration.

Transmission efficiency was determined by the proportion of mosquitoes with virus in the saliva among tested ones (*i*.*e*., surviving females including females unable to disseminate the virus and those able to disseminate). The number of infectious particles per saliva was determined by titration using focus fluorescent assay on C6/36 *Ae*. *albopictus* cells. Saliva samples were serially diluted and inoculated onto C6/36 *Ae*. *albopictus* cell culture in 96-well plates. After incubation at 28°C for three days, plates were stained using hyper-immune ascetic fluid specific to CHIKV as primary antibody. Alexa Fluor 488 goat anti-mouse IgG was used as the second antibody (Life technologies). The lower detection limit of an assay was 2 FFU/saliva.

### Deep sequencing of saliva samples

About 20 saliva samples from each mosquito population infected with CHIKV_SM were collected at day 7 pi, pooled and deep sequenced. Before pooling, saliva were diluted to obtain comparable numbers of CHIKV particles allowing to have an appropriate representation of the overall viral population. Whole genome sequences (excluding the first 19 nucleotides of the 5’UTR, the 3’UTR and the 25 last nucleotides of the second open reading frame) were determined for pooled saliva using the Ion PGM Sequencer (Life Technologies) as described by Rothberg and colleagues [29], and sequence analysis was conducted using CLC Genomics Workbench 6 software. For deep sequencing, a set of four primer pairs (S1 Table) was used to generate amplicons with 3 μL of nucleic acid extract and the Superscript III One-Step RT-PCR Platinum TaqHifi kit (Life Technologies) according to manufacturer’s instructions using the following cycling parameters: 50°C for 30 min, 94°C for 2 min followed by 45 cycles of 94°C for 15 sec, 56°C for 30 sec and 68°C for 4 min. PCR products were verified by gel electrophoresis, and amplicons were purified using Amicon Ultra—0.5 mL 30K kit (Millipore) according to the manufacturer's instructions. For each sample, an equimolar mix of all amplicons was used to build a library and produce the corresponding sequences for the Ion PGM Sequencer according to the manufacturer's instructions. The reads obtained were trimmed: first using quality score and then by removing the primers used for amplification. Reads were mapped to the genome sequence of CHIKV_SM produced following two passages on Vero cells, which was used as a reference. Mutation frequencies (proportion of viral genomes with a specific mutation) at each position were calculated as the number of reads with the mutation compared to the reference divided by the total number of reads at that site. Only substitutions with a mutation frequency ≥ 5% were considered significant for further analysis. The 5% cut-off was considered in our NGS experimental protocol applied to CHIKV plasmid sequence to avoid any background minor variants due to the sequencing method.

### Statistical analysis

All statistical tests were conducted using the STATA software (StataCorp LP, Texas, USA). P-values >0.05 were considered non-significant. Frequencies were compared using Fisher’s exact test and sample distributions with the Kruskal-Wallis test. If multiple Fisher's tests were applied to the same data set, then the significance level for each test was adjusted by the sequential Bonferroni method to accommodate the multiple tests.

## Results

### *Aedes aegypti* from Saint-Martin Island transmit CHIKV_SM and CHIKV_LR with similar efficiency

**Fig 1:**
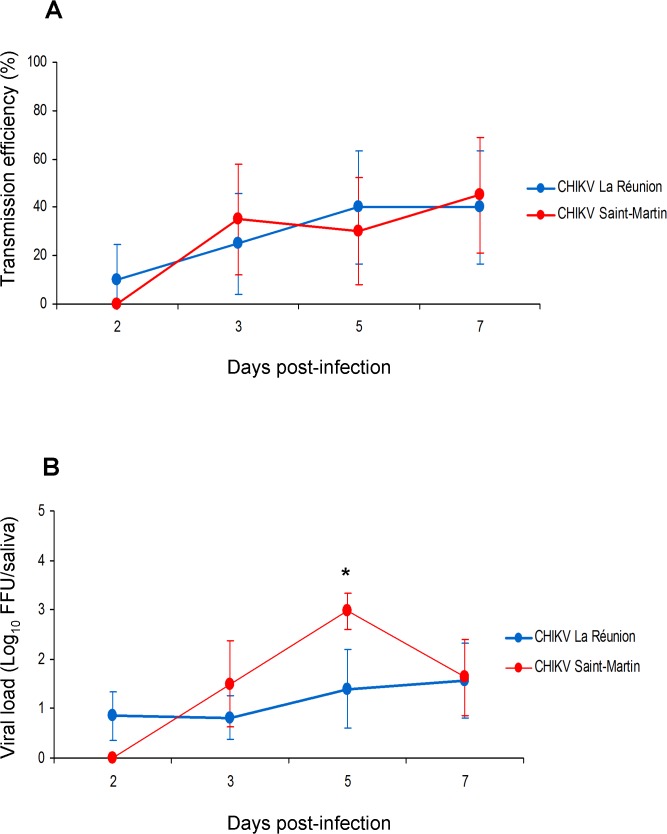
Transmission efficiencies (A) and viral loads (B) in saliva of *Aedes aegypti* from Saint-Martin at different days after infection with CHIKV_LR and CHIKV_SM provided at a titer of 10^6.5^ PFU/mL. Mosquitoes incubated at 28°C were examined at days 2, 3, 5 and 7 to determine the transmission efficiency and the number of viral particles in saliva. Transmission efficiency corresponds to the proportion of mosquitoes with infectious saliva among the tested ones. An asterisk refers to a significant difference (P-value < 0.05). Error bars represent the confidence interval (95%) for transmission efficiencies, and the standard deviation for viral loads.

To characterize the ability of *Ae*. *aegypti* from Saint-Martin to transmit CHIKV_SM, we evaluated transmission efficiencies and viral loads in saliva at days 2, 3, 5, and 7 pi. As a control, we also infected mosquitoes with CHIKV_LR. Our studies indicate that CHIKV_LR could be detected from day 2 pi (transmission efficiency: 10%; viral load: 0.8±0.5 log_10_) and CHIKV_SM from day 3 pi (transmission efficiency: 35%; viral load: 1.5±0.9 log_10_) when provided in blood-meals to *Ae*. *aegypti* SMAR from Saint-Martin. In addition, we found that at a given day pi, no significant difference in transmission efficiencies were detected between the two viruses, CHIKV_SM and CHIKV_LR (P-value > 0.05) (Fig 1A). When examining viral loads in saliva, a similar pattern was obtained except at day 5 pi where the distribution of viral loads in *Ae*. *aegypti* SMAR was significantly higher when infected with CHIKV_SM than with CHIKV_LR (P-value < 0.05) (Fig 1B).

### *Aedes aegypti* from the Americas display similar and moderate transmission efficiency for CHIKV_SM

**Fig 2:**
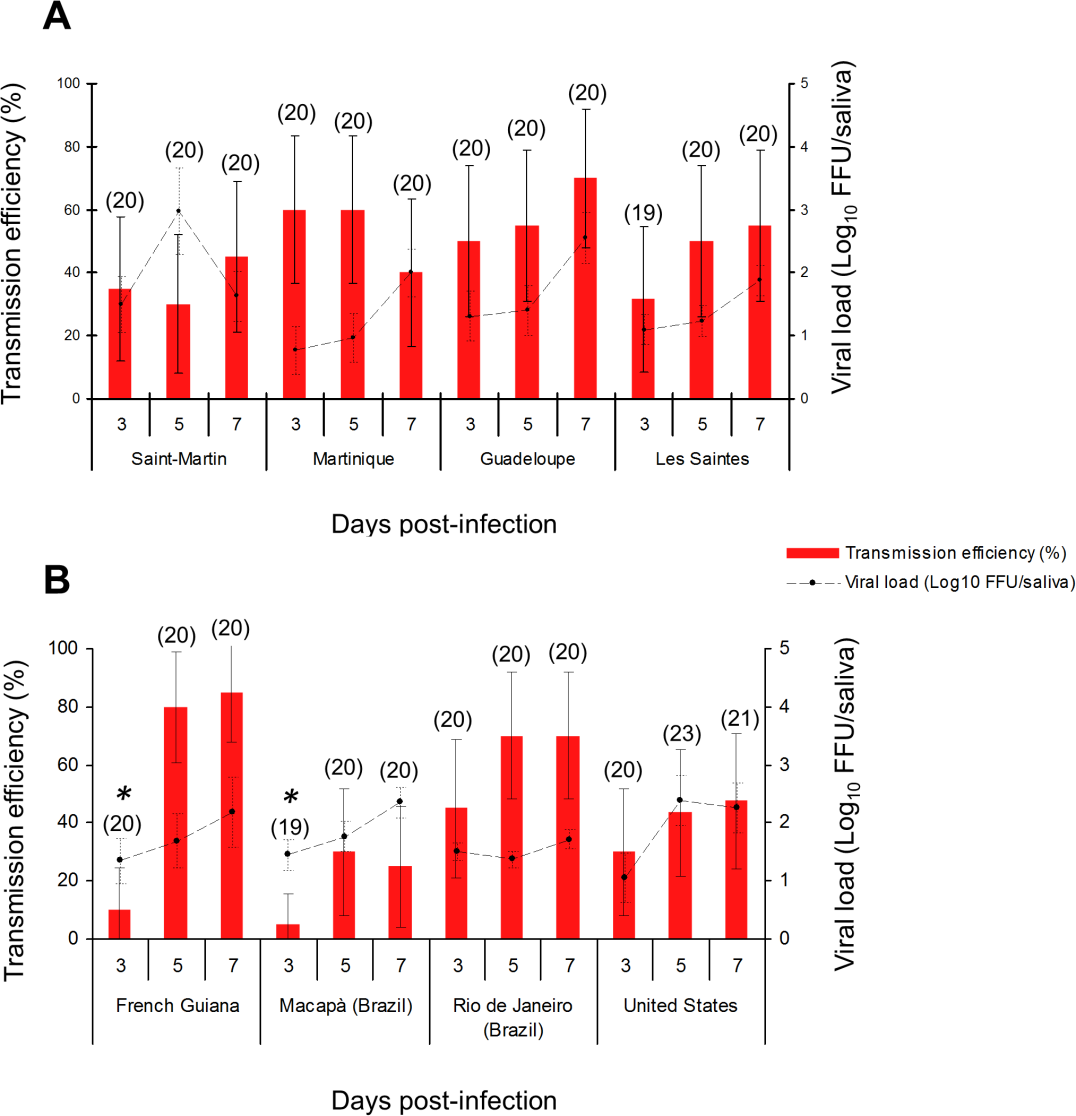
Transmission efficiencies (A) and viral loads (B) of CHIKV_SM in saliva of *Ae*. *aegypti* collected in the Caribbean (A) and the continental America (B). At days 3, 5 and 7 after an infectious blood-meal, 20 mosquitoes per condition were sacrificed for saliva collection and saliva was titrated on C6/36 *Ae*. *albopictus* cells. Transmission efficiency corresponds to the proportion of mosquitoes with infectious saliva among the tested ones. In parenthesis, the number of analyzed mosquitoes. Error bars represent the confidence interval (95%) for transmission efficiencies, and the standard deviation for viral loads.

To evaluate the risk of CHIKV spread throughout the Americas, we compared transmission efficiencies among *Ae*. *aegypti* mosquitoes from localities in the Americas. All mosquitoes were susceptible to CHIKV_SM (Fig 2A and 2B). At each day pi, we did not find any significant difference in transmission efficiencies between mosquito populations (P-value > 0·05), except for mosquitoes from French Guiana and Macapá (P-value < 0.05). At day 3 pi, transmission efficiency was significantly reduced (~10%) in these populations (P-value < 0.05). When examining mosquito susceptibilities to CHIKV_LR, no differences were found between mosquito populations except for *Ae*. *aegypti* from Macapá which displayed low transmission efficiencies not exceeding 25% (P-value < 0.05) (S1 Fig). When examining viral loads, no significant differences were detected regardless of population (P-value > 0.05) (Fig 2A and 2B).

### No differences detected between *Ae*. *albopictus* and *Ae*. *aegypti* from continental America in transmission efficiency of CHIKV_SM

**Fig 3:**
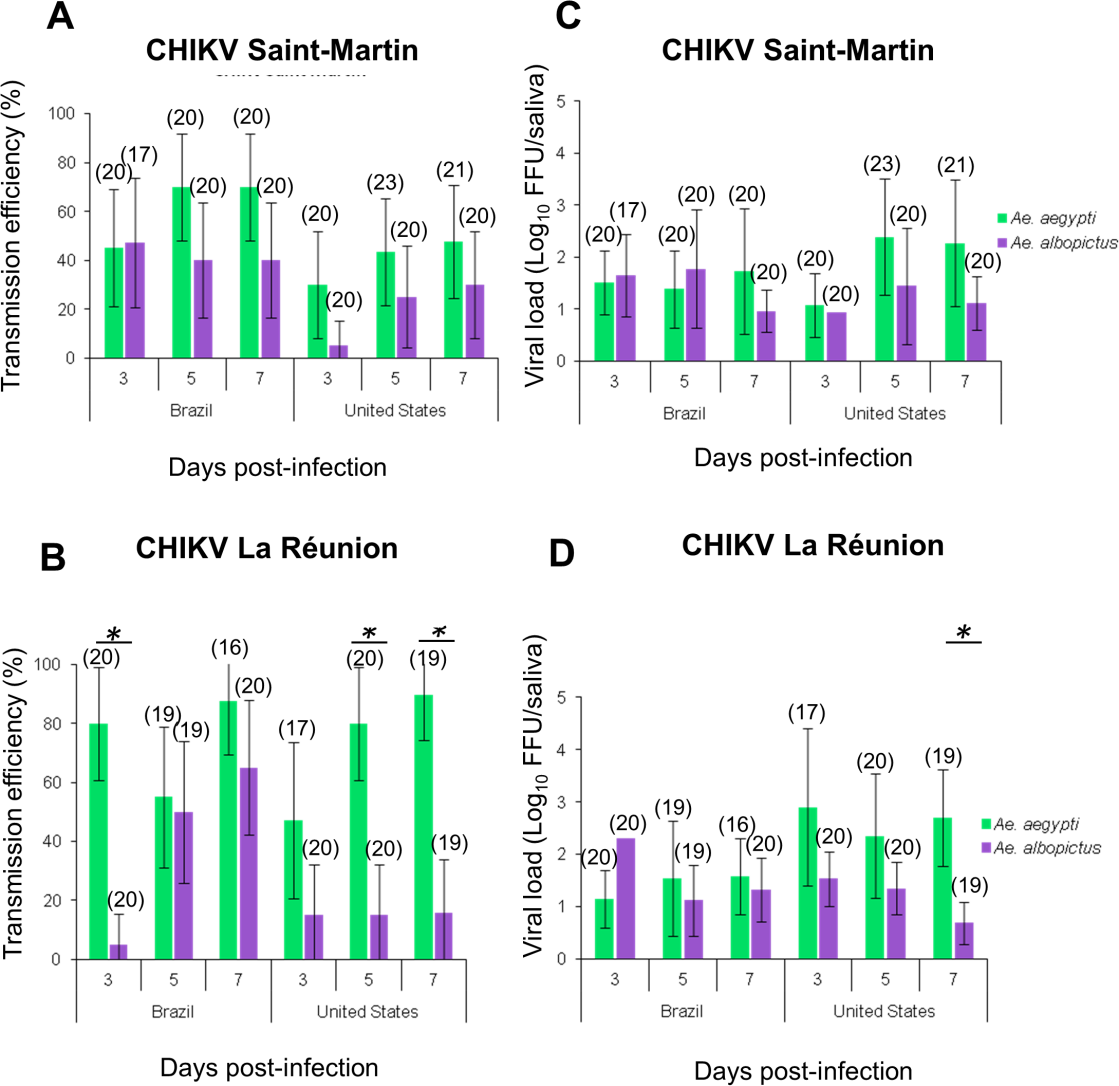
Susceptibility of *Aedes aegypti* compared to *Aedes albopictus* from the same locality when infected with CHIKV_SM and CHIKV_LR and examined at different days after infection. Twenty mosquitoes per condition were processed to determine the transmission efficiency (A and B) and the viral load in saliva at days 3, 5 and 7 after an infectious blood-meal (C and D). Transmission efficiency corresponds to the proportion (%) of mosquitoes with infectious saliva among the tested ones. In parenthesis, the number of analyzed mosquitoes. An asterisk refers to a significant difference (P-value < 0.05). Error bars represent the confidence interval (95%) for transmission efficiencies, and the standard deviation for viral loads.

To characterize the susceptibility of the alternative vector *Ae*. *albopictus* to CHIKV_SM, we compared transmission efficiencies and viral loads in saliva between *Ae*. *aegypti* and *Ae*. *albopictus* collected from two regions (Brazil and the United States). CHIKV_LR was used as a control. Both mosquito species exhibited similar transmission efficiencies for CHIKV_SM at each day pi (P-value > 0.05) (Fig 3A). *Ae*. *aegypti* was significantly more susceptible to CHIKV_LR than *Ae*. *albopictus* at day 3 pi for mosquitoes from Brazil, and at days 5 and 7 pi for mosquitoes from the United States (Fig 3B). When comparing *Ae*. *albopictus* transmission efficiencies between viral strains, differences were only observed for *Ae*. *albopictus* from Rio de Janeiro at day 3 pi (47.06% for CHIKV_LR *versus* 5% for CHIKV_SM). Overall, viral loads were similar (Fig 3C and 3D) except at day 7 pi for mosquitoes from the United States infected with CHIKV_LR (Fig 3D).

### *Ae*. *albopictus* from Southern France transmit both CHIKV_LR and CHIKV_SM at 28°C, but CHIKV_SM transmission is significantly decreased at 20°C

**Fig 4:**
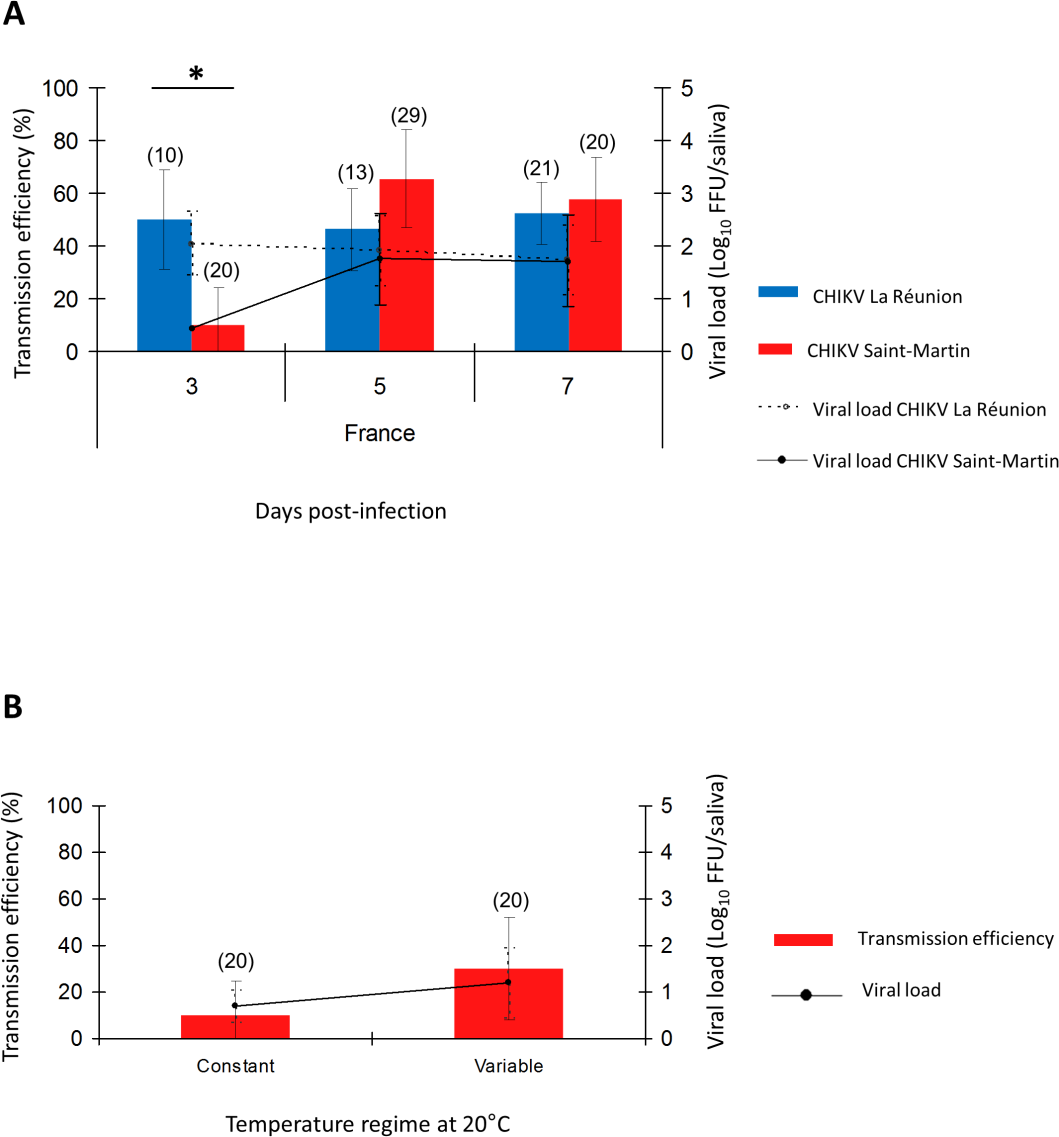
Effect of temperature on CHIKV transmission. *Ae*. *albopictus* from Bar-sur-Loup (France) were infected with CHIKV_LR and CHIKV_SM and incubated at 28°C (A). Mosquitoes infected with CHIKV_SM were also exposed at a constant temperature of 20°C or at variable temperatures mimicking daily fluctuations around an average of 20°C (B). Transmission efficiencies and viral loads in saliva were determined as previously described using 20 mosquitoes per condition. In parenthesis, the number of analyzed mosquitoes. An asterisk refers to a significant difference (P-value < 0.05). Error bars represent the confidence interval (95%) for transmission efficiencies, and the standard deviation for viral loads.

To determine if temperate *Ae*. *albopictus* were more susceptible to CHIKV_SM than to CHIKV_LR, we evaluated transmission efficiencies and viral loads in saliva at days 3, 5, and 7 pi (Fig 4A). We found that transmission efficiency was significantly higher with CHIKV_LR at day 3 pi (P-value < 0.05), while no difference was found between viral strains at days 5 and 7 pi (P-value > 0·05). Viral loads in saliva were not significantly different (P-value > 0.05) at any day pi.

To define if transmission of CHIKV_SM was lowered at a colder temperature, we compared transmission efficiencies and viral loads in saliva at each day pi between temperate *Ae*. *albopictus* incubated at 28°C and at 20°C after oral infection. The virus was detected from day 3 pi when mosquitoes were incubated at 28°C and only at day 7 pi when incubated at 20°C (Fig 4B). At day 7 pi, transmission efficiencies were significantly higher at 28°C (57.5%±7.9) than at 20°C (10%±6.9) (P-value < 0.05) (Fig 4B). When examining viral loads in saliva, we obtained the same pattern with a higher viral load detected in saliva of mosquitoes incubated at 28°C (1.7±0.8 log_10_) than at 20°C (0.7±0.3 log_10_) (Fig 4A and 4B). When mosquitoes were incubated with daily fluctuations of temperature with a mean value of 20°C, transmission efficiencies and viral loads in *Ae*. *albopictus* saliva were slightly increased (Fig 4B).

### More viral genetic diversity of CHIKV_SM is observed after passage through *Ae*. *aegypti* from Saint-Martin

Using pools of saliva from each mosquito populations, mutation frequencies were estimated from nucleotide polymorphisms at each position of the viral genome using the CHIKV_SM produced on Vero cells as reference. Thirty-four nucleotide substitutions (of frequency higher than 5%) were detected throughout the viral genome: 25 in non-structural genes, and nine in structural genes. (Table 2). Notably, we did detect neither E1-A226V nor E1-T98A substitutions, or other described *Ae*. *albopictus*-adaptive mutations in any pool of mosquito saliva. Overall, results were heterogeneous regardless of *Ae*. *aegypti* or *Ae*. *albopictus* populations, with one exception, *Ae*. *aegypti* SMAR from Saint-Martin. While the number of mutations for other mosquito populations ranged from 0 to 6 (median = 3), we detected 13 mutations for the *Ae*. *aegypti* SMAR. In addition, 62% (8/13) of these mutations were synonymous while the proportion of synonymous mutations for other mosquito populations ranged from 0% to 50% (median = 17%). This specific mutation pattern was also associated with a particular distribution in the viral genome: 54% (7/13) of them were located in the structural genes (almost all of them were synonymous) representing 78% of the mutations detected in this region. Only two other mutations were detected in the structural genes of the virus population from saliva of *Ae*. *aegypti* MACA from Brazil. Saliva from *Ae*. *aegypti* SAIN and *Ae*. *albopictus* USA were not analyzed due to technical problems.

**Table 2:** Viral populations in the saliva of mosquitoes used.

nt position	nt change	Gene	aa change	Frequency of reads (%)								
				*Aedes aegypti*							*Aedes albopictus*	
				SMAR	GUAD	MARTI	CAYE	MACA	RIO	USA	RIO	FRA
**330**	G-> A	nsP1	R85H					7.3				
**719**	A-> C	nsP1	T215P						16.6	13.7		
**764**	G-> A	nsP1	G230R						7.6			
**1195**	A-> T	nsP1	Q373H							33.8		
**1359**	T-> C	nsP1	T428I						6.3		9	15.8
**1440**	T-> C	nsP1	L455P		26.2							
**3309**	A-> G	nsP2	Y543C				27.4					8.4
**3424**	C-> T	nsP2	-						18.3			
**3650**	C-> A	nsP2	L657I	5.6								
**3790**	G-> A	nsP2	I703M	90.9								
**4154**	G-> A	nsP3	G27R								38.7	
**4264**	T-> C	nsP3	-									9.4
**4555**	C-> T	nsP3	-	15.9								
**4876**	C-> T	nsP3	-					5.3				
**5300**	A-> G	nsP3	I409V	5.5								
**5439**	T-> C	nsP3	A455V	91								
**5544**	T-> C	nsP3	L490P		9.9							
**5633**	C-> T	nsP3	R520stop		60.2		33.4	6.6	36.4	64		38.9
	C-> A		-				12.8					
**5635**	A-> T	nsP3	-				5.9					
**5878**	A-> C	nsP4	-									8.1
**6219**	C-> T	nsP4	S189F						15.0			
**6460**	T-> G	nsP4	-									5.4
**7018**	G-> A	nsP4	-	86.9								
**7331**	A-> G	nsP4	T560A								83.3	
**7644**	C-> T	capsid	-					83.3				
**8832**	T-> C	E2	-	7.7								
**8922**	T-> C	E2	-	10.1								
**8940**	C-> T	E2	-	9.4								
**9057**	A-> T	E2	-	10								
**9952**	C-> A	6K	L52M	10.1								
**10242**	C-> T	E1	-	9.6								
**10365**	C-> T	E1	-	10.2								
**10875**	C-> T	E1	-					10				

CHIKV_SM produced after two passages on Vero cells was used to infect mosquitoes and as reference genome.

nt, nucleotide; aa, amino-acid; nt; SMAR corresponds to Saint-Martin; GUAD, Guadeloupe; MARTI, Martinique; CAYE, Cayenne; MACA, Macapà; RIO, Rio de Janeiro; USA, United States; FRA, France.

## Discussion

In December 2013, local transmission of CHIKV was reported on Saint-Martin Island in the Caribbean [6]. Although multiple imported cases of CHIKV had been reported in the New World, epidemic spread of the virus did not occur until approximately ten years after its expansion from costal Kenya in 2004 [1]. Surprisingly, it was the Asian genotype of CHIKV which caused this epidemic, as opposed to the more widespread ECSA genotype. Additionally, the mosquito *Ae*. *aegypti* has been implicated as the main vector in this epidemic. Although this combination of mosquito and virus has been documented in sporadic outbreaks [30], our understanding of virus-vector interactions remains limited. Here, we demonstrate that (i) *Ae*. *aegypti* from Saint-Martin Island were able to efficiently transmit both Asian and ECSA genotype CHIKV strains isolated from Saint-Martin and La Reunion respectively, (ii) *Aedes aegypti* from the Americas display similar and moderate transmission efficiency for CHIKV_SM, and (iii) *Ae*. *albopictus* from the Americas were as competent as *Ae*. *aegypti* in transmission of CHIKV_SM. Taken together, our findings highlight the potential for further spread of CHIKV within the Americas as well as a potential role of *Ae*. *albopictus* in this context. Additionally, our findings that European *Ae*. *albopictus* are capable of transmitting both CHIKV_SM and CHIKV_LR raises concerns about the potential for future CHIKV epidemics in Europe.

First isolated in 1952 in Tanzania, CHIKV dramatically expanded its geographic distribution over the last decades, the first wave spreading from Africa to India and Southeast Asia, and the second wave from the coastal Kenya to the Indian Ocean region [9]. In 2004, the ECSA genotype was responsible for the spread of CHIKV beyond its traditional geographic distribution into many tropical regions [31]. In addition, CHIKV strains from the ECSA genotype have previously been identified in Europe: in Italy in 2007 and France in 2010 [14,15]. A common theme for this second wave of expansions is the role of *Ae*. *albopictus* as the primary arthropod vector. This is due in large part to a single amino-acid mutation in the CHIKV E1 glycoprotein (E1-A226V) which increased the vector competence of *Ae*. *albopictus* approximately 50-fold compared to the more traditional vector *Ae*. *aegypti* [32,33]. Second-step *Ae*. *albopictus*-adaptive mutations such as E2-K252Q and E2-L210Q, detected in CHIKV isolated from India in 2007 and 2009 respectively, may also have contributed to the spread and rapid diversification of CHIKV lineages [34],10,24]. Our results contrast with previous findings since we found that *Ae*. *aegypti* was more susceptible to CHIKV_LR than *Ae*. *albopictus* at day 3 pi for mosquitoes from Brazil, and at days 5 and 7 pi for mosquitoes from the United States (Fig 3B). Geographically distant mosquito populations can correspond to genetically differentiated populations presumably causing the differences observed. While previous studies were mainly based on field-collected mosquitoes [32] or laboratory-adapted strains [33], our study reports on transmission by detecting virus in saliva of mosquitoes of the F1 generation.

While the ECSA genotype has continued to spread throughout Southeast Asia [1], the Asian genotype has affected limited regions in the Pacific region with the virus progressing in small jumps from East Asia to the Western Pacific [30,35]. As early as 2007, the potential of CHIKV emergence in the Americas was strengthened by human populations mostly naïve to CHIKV combined with high densities of competent *Ae*. *aegypti* and *Ae*. *albopictus* vectors [1,3]. The threat became reality in late 2013 with the detection of the first autochthonous CHIKV cases on the Caribbean island of Saint-Martin. The virus belonged to the Asian genotype and was closely related to strains from East Asia (Philippines and China) [6]. At the time of writing, local transmission had been identified in 43 countries in the Americas with more than 1,000,000 cases reported (http://www.cdc.gov/chikungunya/geo/index.html). The mosquito *Ae*. *aegypti* has been identified as the main vector in this epidemic, spreading the virus from Saint-Martin, throughout the Caribbean. Here, we showed that *Ae*. *aegypti* SMAR were able to efficiently transmit both ECSA and Asian genotypes of CHIKV at rates similar to *Ae*. *aegypti* from New Caledonia [35]. Deep sequencing reveals that this efficient transmission was not associated with the emergence of viral populations harboring the E1-A1226V and/or the E1-T98A in any *Ae*. *aegypti* or *Ae*. *albopictus* population. Intriguingly, we found a large number of synonymous mutations in the saliva of *Ae*. *aegypti* SMAR seven days after oral challenge with CHIKV_SM (Table 2) while up to three variants were identified in *Ae*. *aegypti* collected from neighboring islands (Martinique and Guadeloupe). This may suggest that bottlenecks induced by mosquito internal barriers (*i*.*e* midgut, salivary glands) were less constraining for the CHIKV_SM in *Ae*. *aegypti* SMAR mosquitoes than in *Ae*. *aegypti* from neighboring islands. It is tempting to propose that CHIKV_SM is naturally well adapted to *Ae*. *aegypti* SMAR, and therefore able to rapidly express a particular mutant spectrum consisting of a majority of synonymous mutations. Thus, CHIKV emergence and rapid spread through the Caribbean is due to a CHIKV_SM well adapted to *Ae*. *aegypti* from Saint-Martin Island, and to the ability of mosquitoes from the West Indies to transmit Asian strains of CHIKV.

Similar to previous epidemics of other vector-borne diseases, CHIKV has expanded outside its traditional range of distribution (and unusually, emerging in temperate regions) following the worldwide expansion of *Ae*. *albopictus*. This vector is an invasive species currently found in temperate and tropical regions [11,13]. Here, we found that regardless of population origin, susceptibility of *Ae*. *albopictus* from the Americas was similar for both the ECSA genotype (CHIKV_LR) and the Asian genotype (CHIKV_SM) at days 5 and 7 pi. Differences were only detected at day 3 pi where, surprisingly, transmission by *Ae*. *albopictus* collected from Rio de Janeiro (Brazil) was 10 times higher with CHIKV_SM (~ 50%) than with CHIKV_LR (~5%) (Fig 3A and 3B), corroborating the threat of CHIKV_SM for this country. However, temperate *Ae*. *albopictus* exhibited the opposite pattern of transmission: with CHIKV_LR, transmission efficiency remained high (~ 50%) from day 3 pi while it reached a similar level only at day 5 pi with CHIKV_SM (Fig 4A). Differences on transmission efficiencies of CHIKV_SM and CHIKV_LR observed between *Ae*. *albopictus* from the Americas and Europe highlight the need for further genetic analysis in order to elucidate the phylogenetic relationships for these mosquito populations.

Since ambient temperature plays a key role in modulating mosquito vector competence for pathogens [21,22], we also incubated temperate *Ae*. *albopictus* at lower temperatures. When infected mosquitoes were incubated at 20°C, corresponding to a mean temperature recorded where local CHIKV transmission was detected in Italy and in southeast France [14,28], the virus was detected very late at day 7 pi in mosquito saliva (transmission efficiency, ~ 10%). When mimicking daily fluctuations of temperature around the mean value of 20°C, transmission efficiency was slightly enhanced though no significant differences were observed (Fig 4B). This result contrasts with our previous findings where *Ae*. *albopictus* from southern France transmitted the ECSA genotype better at 20°C compared to 28°C [17]. This was supported in late October 2014 with the detection of 5 CHIKV autochthonous cases in Montpellier, Southern France (http://www.invs.sante.fr/). Viral isolates from these patients belonged to the ECSA genotype (Leparc-Goffart, personal communication). Despite the hundreds of infected people returning to France from the Caribbean during the summer and later [12], no autochthonous transmission of the imported Asian CHIKV genotype was detected, supporting that transmission is strongly dependent on the mosquito population genetics, the viral genotype and environmental conditions such as the temperature [17]. According to our results based mainly on vector competence, it is crucial for American and European countries to be prepared for more vector-borne disease epidemics. Vector control measures should be triggered very quickly to prevent transmission of the virus by local mosquitoes. With CHIKV_LR and CHIKV_SM, only three days after infection are needed to initiate an outbreak. Although our results show differences in vector competence, other factors (mosquito densities, feeding behavior, mosquito survival rate…s) composing the vector capacity, are needed to assess more accurately the risk of CHIKV transmission.

## Supporting Information

S1 Table Set of primers used for the amplification and sequencing of CHIKV strains. (DOCX)

S1 Fig Transmission efficiencies (A) and viral loads (B) of CHIKV_La Réunion in saliva of *Ae*. *aegypti* collected in the Caribbean (A) and the continental America (B). At days 3, 5 and 7 after an infectious blood-meal, 20 mosquitoes were sacrificed for saliva collection and saliva was titrated on C6/36 *Ae*. *albopictus* cells. Transmission efficiency corresponds to the proportion of mosquitoes with infectious saliva among the tested ones. In parenthesis, the number of analyzed mosquitoes. Error bars represent the confidence interval (95%) for transmission efficiencies, and the standard deviation for viral loads.
(TIF)
