# Supplementary material for: Chikungunya Virus Transmission Potential by Local Aedes Mosquitoes in the Americas and Europe
Source: PLoS Negl Trop Dis. 2015 May 20;9(5):e0003780. doi: 10.1371/journal.pntd.0003780 (PMC4439146; doi:10.1371/journal.pntd.0003780)
Supplement: S1 Table — (DOCX) [file pntd.0003780.s001.docx]

**Table S1. Set of primers used for the amplification and sequencing of CHIKV strains.**

| **Sequence (5’ to 3’)** | **Forward/Reverse** | **nt position** |
| --- | --- | --- |
| ATGGCTGCGTGAGACACAC | Forward 1 | 1 - 19 |
| TGGGACCACTGCCTATCATTTA | Reverse 1 | 3162 - 3183 |
| GACCTTTGACACGTTCCAAA | Forward 2 | 3079 - 3098 |
| GAACCTATCCATTGGTACATC | Reverse 2 | 6491 - 6511 |
| ACACACTACAGAATGTACTGGCA | Forward 3 | 6243 - 6265 |
| CGTACATGAGTGACTRATCTTCCT | Reverse 3 | 8893 - 8916 |
| CAGCACCGTGCACGATTACTGG | Forward 4 | 8792804 - 8813 |
| GCCTGCTAAACGACACGCAT | Reverse 4 | 11277 - 11296 |
