# Supplementary figures and images for: Chikungunya Virus Transmission Potential by Local Aedes Mosquitoes in the Americas and Europe
Source: PLoS Negl Trop Dis. 2015 May 20;9(5):e0003780. doi: 10.1371/journal.pntd.0003780 (PMC4439146; doi:10.1371/journal.pntd.0003780)

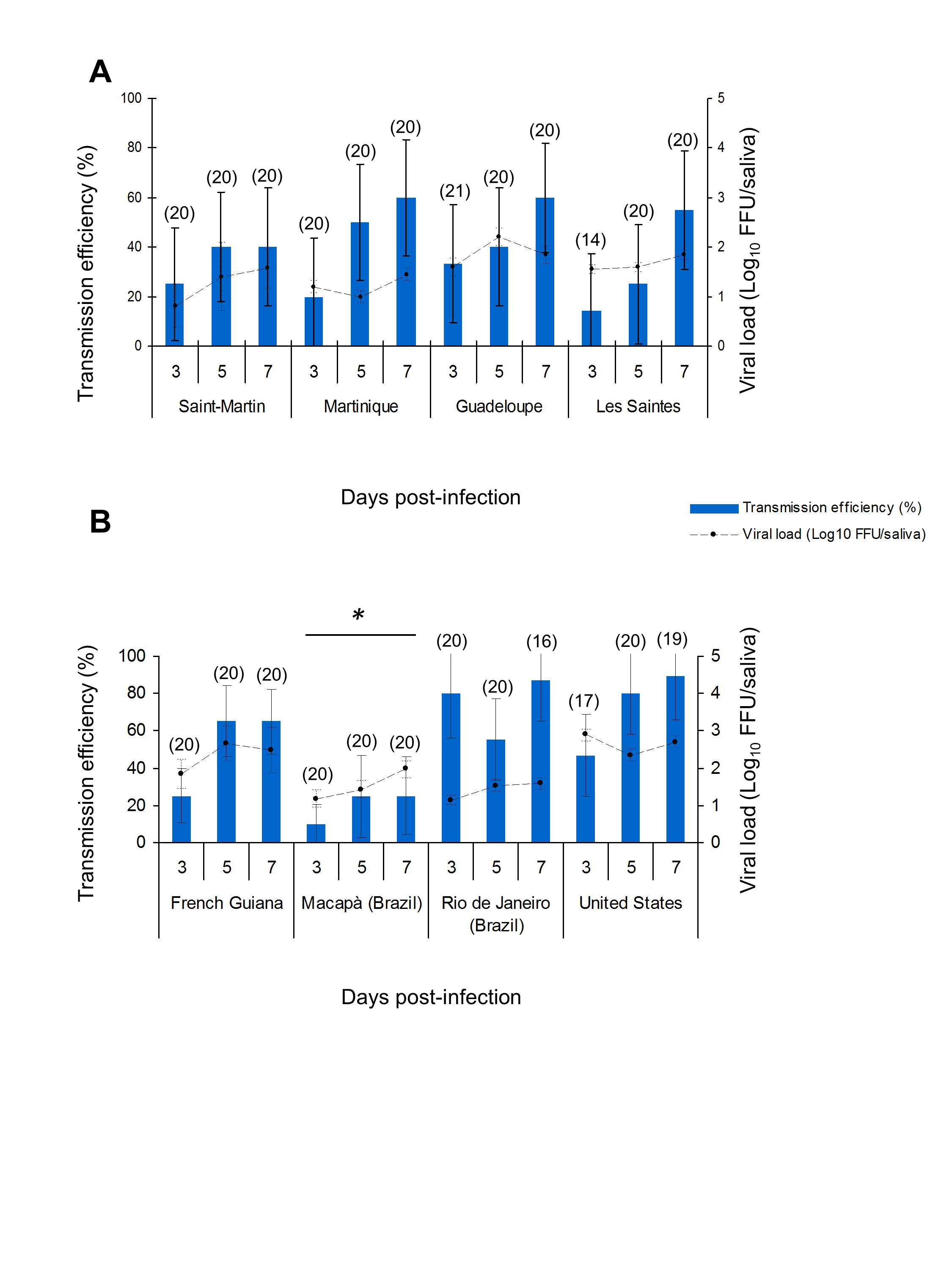

Supplement: S1 Fig — At days 3, 5 and 7 after an infectious blood-meal, 20 mosquitoes were sacrificed for saliva collection and saliva was titrated on C6/36 Ae. albopictus cells. Transmission efficiency corresponds to the proportion of mosquitoes with infectious saliva among the tested ones. In parenthesis, the number of analyzed mosquitoes. Error bars represent the confidence interval (95%) for transmission efficiencies, and the standard deviation for viral loads. (TIF) [file pntd.0003780.s002.tif]
